# Endovascular Management of Aorta-Iliac Stenosis and Occlusive Disease by Kissing-Stent Technique

**DOI:** 10.1155/2016/4035307

**Published:** 2016-01-05

**Authors:** Meng Liu, Fuxian Zhang

**Affiliations:** ^1^Department of Vascular Surgery, Beijing Shijitan Hospital, Capital Medical University, Beijing 100038, China; ^2^Department of Vascular Surgery, Tianjin Hospital, Tianjin 300211, China

## Abstract

Kissing-stenting treatment has been used to treat patients with peripheral artery disease (PAD). However, the long term efficacy of the stenting therapy is not well defined in Chinese PAD patients. To investigate the question, sixty-three PAD patients (37 males and 26 females), aged 66 ± 7.3 years, were analysed in the study. They were featured as claudication (*n* = 45, 71.4%), rest pain (*n* = 18, 28.6%), or gangrene (*n* = 8, 12.7%). In total, 161 stents were applied in aorta-iliac lesions with 2.6 stents for each patient, including 55 self-expanding stents, 98 balloon expandable stents, and 8 covered stents. The success rate of implanting Kissing-stents was 100%. Catheter-directed thrombolysis (CDT) with urokinase was performed in 8 cases (12.7%). The severity of peripheral ischemia was significantly improved, as evidenced by 3.3-fold increase of ankle-brachial pressure index (ABI) after the surgery (*P* = 0.008). One, three, five, and seven years after surgery, the primary patency rate was 87.3%, 77.4%, 71.1%, and 65.0%, whereas the secondary patency rate was 95.2%, 92.5%, 89.5%, and 85.0%, respectively. No in-hospital mortality was recorded. In conclusion, Kissing-stenting technique for aorta-iliac lesions is safe and effective with lower complications. It is beneficial for aorta-iliac occlusions that are longer than 60 mm.

## 1. Introduction

Aorta-iliac stenosis and occlusion contribute substantially to limb ischemia in patients with peripheral artery disease (PAD). Conventional treatments include bypass surgery such as aorta-iliac artery bypass, axillofemoral artery bypass, and bilateral femoral artery bypass [1–3]. It has been reported that the percentage of mortality and complications following bypass surgery was about 3–5% and 8–13%, respectively [4]. In recent years, endovascular technics have been developed to be the alternative therapy to PAD. Accumulated data have documented that both mortality and complications of endovascular intervention with Kissing-Technique were significantly reduced compared to traditional surgery in western countries [5, 6]. Nevertheless, the long term efficacy of Kissing-stents in the treatment of Chinese PAD patients remains inconclusive. In addition, whether endovascular intervention has overcome the difficulty of managing bifurcations is not well defined.

To investigate these questions, 63 PAD patients with clear diagnosis of aorta-iliac bifurcation occlusion were analysed in the study. In total, 161 stents were applied in the aorta-iliac lesions in the PAD patients. The success rate of implantation of Kissing-stenting achieved 100%. After stenting, the patients were followed up to 7 years to evaluate the short term and long term patency rates using Kissing-stent technique.

## 2. Materials and Methods

### 2.1. The Studying Subjects

PAD patients (*n* = 63) with a mean age of 66 years who were hospitalized between April 2007 and April 2014 were included in the study. Arteriosclerosis was diagnosed in all patients. Among them, there were 39 smokers (61.9%), 51 cases of hypertension (81.0%), 38 cases of diabetes (60.3%), 30 cases of hyperlipidemia (47.6%), 34 cases of coronary artery disease (54.0%), and 18 cases of cerebrovascular disease (28.6%). All 63 patients were evaluated by duplex ultrasonography, magnetic resonance (MR) angiography, computerized tomography, and ankle-brachial pressure index (ABI) prior to operation. The study received ethical approval from the competent Institutional Review Boards of Capital Medical University and was performed in accordance with relevant guidelines and regulations in Capital Medical University. All participants provided written informed consent.

All the patients met the criteria of using Kissing-stenting treatment as the following: (1) vascular color Doppler ultrasound showed hypoechoic or admixture-echoic signs at the occlusion sites in common iliac artery occlusions despite absence of severe calcification; (2) no anchoring (landing zone) occurred at aorta-iliac junction or proximal position in common iliac artery for PTA and stent placement; and (3) catheter or the guide wire may pass the segments of aorta-iliac occlusive disease. In parallel, the severity of occlusion was further confirmed by digital subtraction angiography (DSA), indicating 42 patients with no proximal iliac stump and 21 patients with short proximal iliac stump (≤1 cm).

### 2.2. Catheter-Directed Thrombosis prior to Stenting

In order to achieve higher endovascular angioplasty success rate and favorable patency with low complication rate, preoperative catheter-directed thrombolysis (CDT) with urokinase was administered through a Unifuse catheter (Uni^*∗*^Fuse Infusion Catheter; AngioDynamics, Queensbury, NY) in patients with long-segment aorta-iliac occlusion (≥60 mm). A total of 750,000 U of urokinase diluted in a 50 mL saline solution was administered to the patients via left brachial artery once per day for 2 days. Heparin (400–600 units/hour) was continuously infused into the patients through the sheath. Plasma level of fibrinogen and activated partial thromboplastin time were examined.

In the study, the covered stent was purchased from Gore VIABAHN (Newark, Delaware, USA) or Fluency Plus (Bard Inc., Germany); the self-expanding stents were from Medtronic (Minnesota, USA) or Boston Scientific (Boston, USA); and the balloon expandable stents were from INVATEC (INVATEC Inc., Minnesota, USA).

### 2.3. Endovascular Angioplasty by Kissing-Stenting Technique

All Kissing-stents were deployed from the proximal end in the aorta, that is, 1–4 cm above the native aortic bifurcation. All self-expanding stents were to be dilated to reach the diameter of the normal vessel, according to angiogram prior to the treatment. In 19 patients without palpable pulses in CFA, the Kissing-stents were entered through the left brachial artery. After placing the stents, the artery was further reinforced by a balloon with the diameter of 4 mm to enhance blood perfusion. For the rest of the patients, guide wire was applied percutaneously following bilateral CFA punctures. The size of stent and balloon was determined according to the reference vessel diameter in the vicinity of the lesion and the contralateral iliac artery.

After stenting, to prevent thrombosis, the patients were prescribed oral intake of 100 mg aspirin and 75 mg clopidogrel daily for 8 weeks and then lifelong monotherapy with aspirin.

### 2.4. Evaluations of Kissing-Stenting Surgery

The patients were followed up to 7 years (interquartile range, 3–84 months). The primary and secondary patency rates were recorded. Technical success was defined as residual stenosis less than 30%, a pressure gradient less than 5 mm Hg, and the increase of ABI greater than 0.1.

Loss of primary patency was diagnosed when ABI index is above 20% or restenosis above 50%. In this situation, a secondary intervention was given to maintain arterial patency.

### 2.5. Statistical Analysis

Statistical analysis was performed by SPSS software (Version 13.0, United Kingdom). Continuous variables are presented as means and standard deviation and proportional data are presented as number and percentage. Unpaired *t*-test was used to compare ABI index before and after the stenting treatment. The Kaplan-Meier method was used to estimate primary and secondary patency rates. Statistical significance was defined as *P* value less than 0.05.

## 3. Results

### 3.1. General Characterization of PAD Patients

All 63 patients had the clinical symptoms of ischemic limbs, among which 25 patients had critically ischemic limbs. Claudication occurred in 45 (71.4%) patients. Mild claudication is defined as walking distance less than 200 meters whereas severe claudication is defined as walking distance less than 30 meters. Among 45 patients with claudication, there were 27 patients (42.9%) with mild claudication and 18 patients (28.6%) with severe claudication and the average walking distance was 88 ± 2 meters. Moreover, all the patients with severe claudication were also featured as rest pain and 8 of them had suffered from toe gangrene. The lesion length of occlusion in aorta-iliac artery or iliac artery was 6.0 ± 4.9 cm. The averaged ABI index was 0.28. The general characteristic of the patients is shown in Table 1. Eight patients had occlusions in aorta-bilateral iliac artery with stenosis in SFA, 12 patients had occlusions in bilateral common iliac artery, 9 patients had occlusions in left common iliac artery with stenosis at bilateral SFA, and 17 patients had occlusions in right common iliac artery. The patients were categorized according to the stratification of Trans-Atlantic Inter-Society Consensus-II (TASC II) [7].  Table 2 illustrated the standardised TASC II stratification. The number of patients in different TASC types and the number of implants in the patients were summarized in Table 3.

### 3.2. Short Term Assessment of Kissing-Stenting Treatment

Prior to operation, CDT with urokinase was used to improve perfusion. In all patients, Kissing-stents were placed successfully with restoration of the patency of aorta-iliac bifurcation segments and the technical success rate was 100%. In total, 161 stents were applied in bilateral common iliac arteries, which included 55 self-expanding stents, 98 balloon expandable stents, and 8 covered stents. In more detail, 5 stents were applied in 8 patients, 3 stents in 17 patients, and 2 stents in 38 patients. After CDT 3 patients originally presenting TASC II D improved to TASC II B, 3 patients improved from TASC II C to TASC II B, and 2 patients improved from TASC II D to TASC II A, separately.

**Figure 1:**
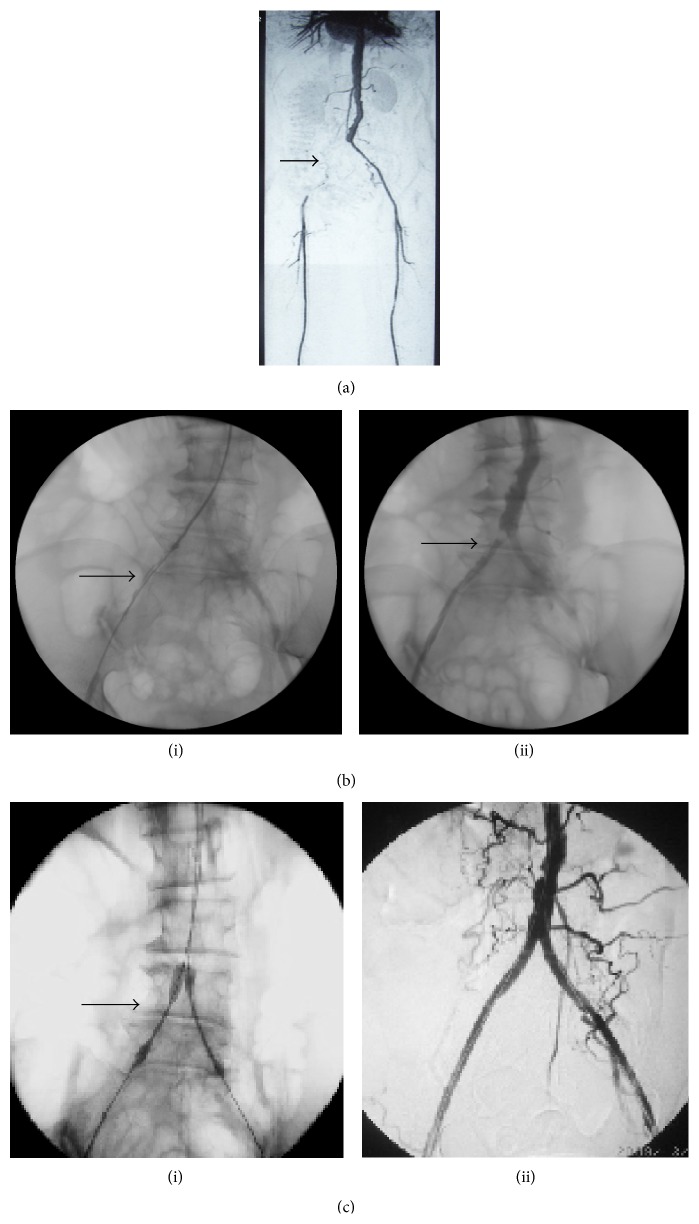
Case (1): stenting for a female PAD patient of 69 years. (a) CTA showed severe right iliac artery occlusive lesions about 12 cm (arrow). (b) (i) Thrombolysis by catheter (arrow). (b) (ii) Two days later, angiogram showed right iliac artery occlusive lesion changed from TASC II D to B (arrows). (c) (i) Endovascular angioplasty by Kissing-stenting (arrow). (c) (ii) Excellent result at right iliac artery.

**Figure 2:**
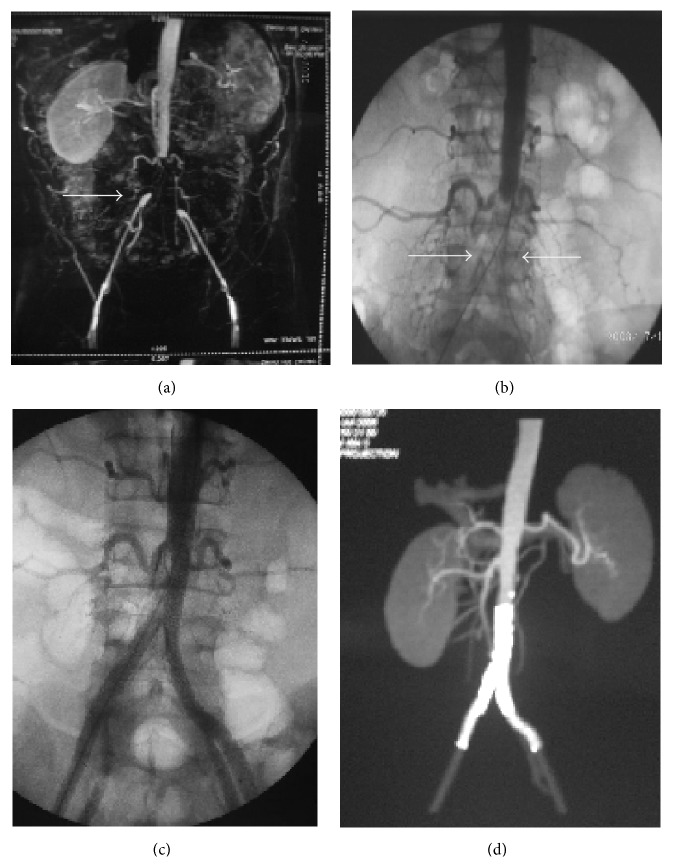
Stenting for a male PAD patient of 46 years. (a) CTA showed aorta-iliac artery occlusion lesions (arrow). (b) Two guide wires pass to aorta-iliac artery from bilateral femoral artery (arrows). (c) After dilation by balloon, Kissing-stents placement involving the distal aorta and bilateral common iliac is performed. (d) Excellent result.

Shortly after the surgery, the stents occluded in 2 patients due to thrombosis and thus they were treated with CDT immediately. Six months after the surgery, stent thrombosis occurred in 7 patients who did not take aspirin regularly after the initial intervention. In these patients, 3 had hematoma, 2 had stroke, 1 had worse renal function, and 1 had brachial pseudoaneurysm. CDT was repeated in these patients to alleviate the thrombosis. The overall complication rate was 14.3% (Table 4). No in-hospital mortality was recorded.  Figure 1 demonstrated a female patient who was featured as severe right iliac artery occlusive lesion and thrombolysis by the catheter during the operation. Kissing-stenting treatment resulted in significant improvement flow in the lesion artery.  Figure 2 displayed another representative treatment of a male PAD patient before and after Kissing-stenting angiography.

### 3.3. Long Term Assessment of Kissing-Stenting Treatment

All the patients were followed up to 7 years to evaluate the efficacy and safety of the stenting therapy. The ABI index increased 3.3-fold by Kissing-stenting treatment (0.28 ± 0.23 versus 0.75 ± 0.18; *P* = 0.008). All patients had complete alleviation of the claudication or rest pain symptoms, the foot ulcers cured, and the average hospitalization days were about 7 days. Overall, the primary patency rate was 87.3%, 77.4%, 71.1%, and 65.0%, and the second patency rate was 95.2%, 92.5%, 89.5%, and 85.0% at 1 year, 3 years, 5 years, and 7 years, respectively.

## 4. Discussion

In 1964, Dotter and Judkins first reported successful interventions in 9 patients who could not accept the traditional surgical operation and were subjected to limb amputation [8]. Nearly two decades later, Kissing-Technique has been used to treat peripheral ischemia, which includes Kissing balloon angioplasty and Kissing-stents [9]. Till present, a number of individual cases have demonstrated the efficacy of the stenting technique. In 2002, Haulon et al. reported 89.5% patency rate at 1 year and 79.4% patency rate at 3 years following stenting [10]. Likewise, Houston et al. illustrated that the primary patency rate was about 68% and the second patency rate went up to 86% after 10 years of Kissing-stenting [11]. Similarly, Björses et al. reported that aorta-iliac Kissing-stents were an alternative treatment to conventional surgery for TASC A–D lesions [12]. The procedure has low mortality and morbidity with good patency at 3 years after stenting. Although Kissing-stenting is a promising tool for the treatment of PAD, more efforts are needed to obtain better long term patency rate. Nevertheless, the efficacy of Kissing-stenting in Chinese PAD patients was not well defined.

The main findings of this study are that Kissing-stenting success rate was 100% and ABI was 3.3-fold improved (*P* < 0.01) 7 years after stenting in the Chinese PAD patients. The primary patency rates were 87.3%, 77.4%, 71.1%, and 65.0%, respectively, after 1, 3, 5, and 7 years of stenting. The secondary patency rate reached 95.2%, 92.5%, 89.5%, and 85.0%, respectively, at 1, 3, 5, and 7 years following stenting. Thus, our data suggest that Kissing-stenting technique for aorta-iliac lesions is safe and effective for long-segment aorta-iliac occlusions. In the literature, Aihara et al. reported that the primary patency after 1 and 5 years of stents was 87% and 73%, respectively, in the treated PAD patients (*n* = 190) [13]. In the meanwhile, Pulli et al. reported that the primary patency rate after 5 years of stents was 82.4% and the secondary patency rate was 93.1% in the patients with iliac artery occlusion (*n* = 109) [14]. Taken together, our data indicate that the long term efficacy of Kissing-stents in the treatment of PAD patients could be comparable between Chinese and western countries.

During endovascular procedures, how to pass through the occlusive lesions was a crucial step in the past and got almost resolved now. However, embolism or occlusion in the contralateral common iliac artery may occur due to shedding of atherosclerotic plaques or thrombotic material during PTA or stenting in unilateral iliac artery in patients with no anchoring (landing zone) or a short stump (≤1 cm) in the aorta-iliac junction or proximal common iliac artery. Thirty days after stenting, reendothelisation in aorta appears in the place where the stents are implanted.

Hereby, we summarized our experience as follows: (1) balloons and stents should be selected in the same diameter with the same length and from the same manufacturer. The diameter of stenting could be oversized to 1.1-fold. (2) The Kissing-stents ought to be placed with the proximal ends in the aorta to the same level and then dilated simultaneously. (3) Self-expanding nitinol stents are the primary choice for patients with long and tortuous lesions (≥60 mm) without heavy calcification and postdilated to the normal vessel diameter. (4) Balloon expandable stents are applied to shorter lesions (≥60 mm) with or without calcification, sometimes to reinforce self-expandable stents if necessary. (5) Covered stents are suggested to treat patients with predicted high rupture risk or with heavy calcification or residual thrombus.

There are several limitations in this study: (1) this is a single-center study with 63 patients in total. Thus the number of study subjects is limited; (2) in the study, we did not categorize the PAD patients on the status of thrombosis. Therefore, it is not clear whether the different type of thrombolysis (acute, subacute, and chronic thrombosis) might have an impact on stenting treatment.

To date, thrombolytic therapy has been accepted as the optional initial treatment for acute limb ischemia but occasionally used to treat patients with chronic limb ischemia. Our experience is to apply CDT with urokinase from brachial access in patients with long-segment aorta-iliac occlusion (≥60 mm) to improve perfusion [15]. We injected urokinase 750,000 units per day for two days and continuous infusion of heparin (400–600 IU per hour) so that the activated partial thromboplastin time was maintained two times higher than normal range. We achieved a higher endovascular angioplasty success rate with no serious complications. Although catheter-directed thrombolysis cannot completely dissolve a clot, it may decrease the complexed surgical procedure and reduce hospitalization time and cost [16]. In addition, we believe that there may be coexistence of stable thrombus and unstable thrombus in the segments of occluded artery. CDT could help to decrease the unstable thrombus load before angioplasty and/or stenting. Therefore, it allows target-specific treatment and decreases the occurrence of embolization by removal of the relatively unstable thrombus components and improvement in the runoff status.

## 5. Conclusions

In conclusion, endovascular management for aorta-iliac stenosis and occlusive disease by Kissing-stenting technique is safe and effective and can raise the endovascular operation success rate with lower complications. Preoperative CDT with urokinase is effective for long-segment aorta-iliac occlusions (≥60 mm).

## Figures and Tables

**Table 1:** General characterization of the studying subjects.

Parameters	PAD patients
*N*	63
Males (%)	37 (63%)
Age (year)	66 ± 7.3
Smoker (0, 1)	39 (61.9%)
*Disease (%)*	
Hypertension (0, 1)	51 (81.0%)
Diabetes (0, 1)	38 (60.3%)
Hyperlipidemia (0, 1)	30 (47.6%)
Coronary heart disease (0, 1)	34 (54.0%)
Cerebrovascular disease (0, 1)	18 (28.6%)
*Medications*	
Aspirin (0, 1)	63 (100.0%)
Clopidogrel (0, 1)	51 (81.0%)
Statins (0, 1)	37 (58.7%)
*Features of PAD*	
Walking distance (m)	88 ± 2 7
Rest pain (0, 1)	18 (28.6%)
Claudication	45 (71.4%)
Mild	27 (42.9%)
Severe	18 (28.6%)

**Table 2:** TASC II classification of aorta-iliac lesions.

Type	Criteria
A	(i) Unilateral or bilateral stenosis of CIA (ii) Unilateral or bilateral single short (≤3 cm) stenosis of EIA

B	(i) Short-segment (≤3 cm) stenosis of infrarenal aorta (ii) Unilateral CIA occlusion (iii) Single or multiple stenosis (3–10 cm) involving the EIA not extending into the CFA (iv) Unilateral EIA occlusion not involving the origins of internal iliac or CFA

C	(i) Bilateral CIA occlusions (ii) Bilateral EIA stenosis (3–10 cm long) not extending into the CFA (iii) Unilateral EIA stenosis extending into the CFA^*∗*^ (iv) Unilateral EIA occlusion that involves the origins of internal iliac and/or CFA^*∗*^ (v) Heavily calcified unilateral EIA occlusion with or without involvement of origins of internal iliac and/or CFA^*∗*^

D	(i) Infrarenal aorta-iliac occlusion^*∗∗*^ (ii) Diffuse disease involving the aorta and both iliac arteries requiring treatment (iii) Diffuse multiple stenosis involving the unilateral CIA, EIA, and CFA^*∗*^ (iv) Unilateral occlusions of both CIA and EIA (v) Bilateral occlusions of EIA (vi) Iliac stenosis in patients with AAA requiring treatment and not amenable to endograft placement or other lesions requiring open aortic or iliac surgery^*∗∗*^

^*∗∗*^Endovascular treatment is not intended for these lesion types. ^*∗*^Lesions involving CFA with severe stenosis are excluded in these types. TASC II, Trans-Atlantic Inter-Society Consensus-II; CIA, common iliac artery; EIA, external iliac artery; CFA, common femoral artery; AAA, abdominal aortic aneurysm.

**Table 3:** Lesion and procedure characteristics.

Characterization	Frequency (%)
Lesion type	
TASC II A	0 (00.0%)
TASC II B	25 (39.7%)
TASC II C	21 (33.3%)
TASC II D	17 (27.0%)
Number of stents implanted (per patient)	
5 stents	8 (12.7%)
3 stents	17 (27.0%)
2 stents	38 (60.3%)
Self-expanding stents	55 (34.2%)
Balloon-expanding stents	98 (60.9%)
Covered stents	8 (5.0%)
Catheter-directed thrombosis	8 (12.7%)

**Table 4:** Frequency of complications.

Complications	Number (%)
Total	9 (14.3%)
In-stent thrombosis	2 (3.2%)
Hematoma	3 (4.8%)
Stroke	2 (3.2%)
Worsening renal function	1 (1.6%)
Brachial pseudoaneurysm	1 (1.6%)
